# Chemistry in Its Relations to Physiology and Medicine

**Published:** 1861-11

**Authors:** 


Art. IX.—*Chemistry in its Relations to Physiology and Medicine.* By Geo. E. Day, M.A., M.D., etc. With five plates, containing numerous engraved illustrations. 8vo., pp. 527. London: Hippolyte Baillière, 1860.

In the preface of the work of Prof. Day we find a candid acknowledgment of the sources from which the author has derived much of the information contained in it. The volume is a fair summary of various opinions, well put together and ably arranged, with a view to facilitate investigation into the chemical physiology of the various organs. The author divides the subject-matter of the volume into three great heads or departments:—

1. The organic substrata of the human body.
2. The chemistry of the animal juices and tissues.
3. The great zoo-chemical processes.

Most of the illustrations, which are all good, are credited to Funke's Atlas der Physiologischen Chemie. A great deal of important information is contained in the different chapters, which we regret we cannot more fully notice; but much of it must lie hidden to the casual reader, from the fact that no index points out the path to it. It is rarely now-a-days that we have to call attention to such a deficiency.

